# Conceptual Frameworks in Neurology Education Research

**DOI:** 10.1212/NE9.0000000000200176

**Published:** 2024-12-30

**Authors:** Andres Fernandez, Jeffrey B. Ratliff, Stefano Sandrone, Subha Ramani

**Affiliations:** 1 Department of Neurology, Thomas Jefferson University, Philadelphia, PA;; 2 School of Health Professions Education, Maastricht University, the Netherlands;; 3 Department of Brain Sciences, Faculty of Medicine, Imperial College London, United Kingdom; and; 4 Department of Medicine, Brigham and Women's Hospital, Harvard Medical School, Boston, MA.

## Abstract

This article discusses conceptual frameworks in neurology education research. We provide practical case examples highlighting the conceptual framework development process for different research scenarios in neurology education. We explore the steps that shape the conceptual framework process, including defining the educational problem, exploring the scholarly conversation on the topic of study through understanding the literature and outlining its gaps, constructing research questions, selecting the theoretical frameworks that underpin the study, and choosing the appropriate methodology and methods for the research questions. We argue that having a clear conceptual framework is fundamental to a rigorous education research study.

## Introduction

Conceptual frameworks represent ways of thinking about a problem or a study^1^ and provide the justification for why a given study should be conducted.^2^ In essence, they articulate the rationale for a study. Specifically, the conceptual framework argues why the study is important, outlines what the study aims to do, and describes how the study aims will be achieved.

The ability to describe why we embark on an educational research project and how to communicate the topic's importance beyond the local context are fundamental aspects of educational scholarship. Although ideas for educational studies abound in neurology, careful consideration must be given to how those ideas are translated into impactful educational research projects.

A clear description of the rationale for neurology education research studies will allow the field to move beyond descriptive studies of educational interventions (e.g., showing that a curriculum was well received by students or that it resulted in an improved post-test score for a given topic) and focus on more foundational questions, such as why and how the educational intervention works or in what way does the learning happen.

This article will discuss conceptual frameworks and provide practical examples centered around neurology education to introduce the reader to this foundational educational research concept.

## What Are the Initial Steps to Consider When Embarking on Neurology Education Research?

There are important early steps to take in building arguments to support the rationale when embarking on an education research study (Table). These steps facilitate the formulation of the study's conceptual framework. From a practical standpoint, the conceptual framework should be described in the introduction and methods sections of the article.

**Table:** Questions to Address to Support the Rationale to Embark on an Education Research Study

**Why is the study important?**
• Define a clear problem statement based on the phenomenon (the problem) being studied and the paradigmatic orientation (assumptions about the phenomenon, e.g., postpositivist vs constructivist paradigmatic orientation)
• Perform a literature review to situate the problem within the broader scientific conversation on the topic, outlining the knowledge gaps and the arguments supporting the relevance of studying the problem
**What does the study aim to do?**
• Define the study aims and objectives
• What is/are the research question(s)? (The research questions determine the focus of the study)
• What is/are the theoretical framework(s) underpinning the study?
**How will the study aims be achieved?**
• What are the methodological choices that align with the research question(s)? (e.g., choosing a qualitative vs a quantitative methodology)
• What are the methods that best align with the research question(s)? (e.g., choosing semistructured interviews vs surveys)

There are many useful approaches to guide educational researchers in describing their conceptual framework; here, we focus on the problem-gap-hook heuristic.^3^ Such a heuristic helps organize a study's rationale by briefly describing an educational problem, summarizing the available literature and knowledge gaps in the area of study, and making the arguments for why narrowing the gap is important. This approach calls for considering the context of a broader scientific conversation we are trying to join and contribute to with the research study. Knowing what is being discussed is essential to understand how to move the conversation forward.

Below are 3 practical examples of how to apply a conceptual framework in different research scenarios commonly encountered in neurology education settings. The examples start with identifying an educational topic of research, which typically emanates from observations during day-to-day educational activities the researchers are involved in through their work. After the researcher identifies a specific topic, the first step is to clearly state the problem that is being studied and the reasons for its importance. A problem statement is not a description or definition of an educational concept but focuses on an important and specific educational problem that warrants further exploration, examination, or intervention (problem). The second step is to review the literature on this topic to determine whether there are gaps, unanswered or partially answered questions that align with the local problem (gap). Next step will be to identify and apply an appropriate conceptual framework that communicates the researchers' purpose, approach, and paradigmatic orientation. This could include using a theory, an established model, guidelines, or best practices, any of which could provide the foundation underpinning the study. The researchers must then make the argument that their research will indeed fill a knowledge gap and advance the field (hook). Finally, the study aims and questions or hypotheses evolve through a combination of the problem statement, the gap, and the conceptual framework. As a point of distinction, theoretical frameworks relate to the use of theory to inform a given study and conceptual frameworks relate to the overall arguments made justifying the study^2^ (these arguments can include how a given theory/theories or models would be used in the study).

## Case 1: Feedback

### Case Vignette

A Neurology Residency Program Director (PD) meets with residents in her program at the end of the academic year to discuss potential changes to improve resident education. The residents reflected on their experience receiving feedback and identified a desire to improve the quality of the feedback they receive from the supervising faculty at the end of their rotations. The PD wonders if there might be a broader research opportunity to find a solution for the specific problem at her institution. More specifically, this project can focus on issues related to resident feedback in the local context, yet the results could provide insights for other neurology programs designing feedback initiatives for their residents.

### Conceptual Framework

#### Define the Educational Problem

The first step of the framework-building process has been formulated: the residents identified a need to improve feedback quality based on their lack of satisfaction with their faculty feedback. The problem to address in this case is the perceived low quality of feedback. When thinking about the phenomenon of feedback between supervising faculty and physician trainees, the PD quickly realizes that effective feedback is an inherently complex and dynamic interaction. There is no single recipe for what constitutes effective feedback that can apply to all contexts. Therefore, some elements of her framework should align with assessing the quality or effectiveness of feedback, while acknowledging that the elements she chooses may differ from other projects examining feedback.

Her other insight is that the feedback problem could be framed in multiple ways because there are many potential research questions around feedback. She realizes that feedback is multifaceted. Some aspects of feedback analysis may focus on the content or setting for the delivery of feedback, such as its verbal content (what information is conveyed by the feedback?), how it is communicated (what communication strategies are used to convey that information?), or the context in which the conversation is situated. Other aspects may focus on the experience of the recipient or provider, such as the readiness of the learner to receive feedback, faculty attitudes toward feedback (how do the faculty experience the process of giving feedback?), emotional responses to feedback on either side, or the ability of the learner to understand what was communicated. Feedback may be examined by its process, outcomes, or impact by asking questions such as perceptions around how or why feedback is effective or not effective. Such questions can be addressed by examining the behavioral changes induced within its recipient (did the learner act differently as a result of the feedback?) or the personal response of the recipient (what impact did the feedback have on the learner's professional identity formation?). These are all potential avenues to be considered while defining a clear problem statement for the research study. Narrowing the scope of the question from “a research project on feedback” to “a research project on a specific aspect of feedback” helps better define what she intends to study.

The PD then considers the relevance of her problem by asking why she should conduct a study on feedback involving neurology residents in the first place. She plans her literature search with the help of her institution's librarian. She also discusses with her neurology PD peers in the American Academy of Neurology (AAN)'s Consortium of Neurology Program Directors (CNPD) whether there are issues around feedback encountered at their residency programs (to assess the relevance and potential transferability of her study findings).

#### Explore the Scholarly Conversation

A potential research project on feedback involves joining a broader scientific conversation and filling a gap in the literature while staying relevant to the local problem. Finding a gap in the literature requires a careful search and review of the studies in the literature around the topic of interest (to determine what is the existing body of knowledge that the study will contribute to). The PD conducts the planned literature search to understand the current state, available evidence, and potential gaps and to understand the theories and theoretical frameworks used by other educators to study feedback. This step helps her move beyond writing a study about a specific problem for the residents at her institution with a narrow scope of interest for others. It allows for framing the research question in a broader context of feedback-related scholarship.

Upon examining the different types of questions and theoretical frameworks researchers have used to study feedback, she has more clarity on the research question she wants to ask for her study.

#### Construct the Research Question(s) and Select the Theoretical Framework(s)

After defining the problem and its relevance and reviewing the articles from her literature search, she decided the study would focus on neurology residents' perceptions of feedback informed by the theoretical framework of educational alliance. Theoretical frameworks define the use of a theory or theories to ground the study.^2^ In this case, the elements of the educational alliance framework will shape the way the research study will be designed and interpreted.

The educational alliance^4^ framework borrows from the therapeutic alliance framework in psychotherapy that states that behavioral change in a patient undergoing psychotherapy is influenced by 3 key components of the therapist-patient relationship: mutual understanding of the therapy's purpose; agreement about how to reach a goal; and the patient's liking, trusting, and valuing of the therapist. Thus, the analogous components of an educational alliance may focus on the learner's perception of the purpose of the feedback, agreement on a specific learning goal during the feedback conversation along with action plans to achieve the goal, and the relationship qualities between the learner and supervisor.

#### Choose the Appropriate Methodology and Methods for the Research Question(s)

The PD formulates a specific research question: what are the perceptions of neurology residents regarding the purpose of the feedback they receive? She plans to investigate the topic guided by the theoretical framework of educational alliance and continues to build the rationale for her study by choosing which methodology and methods will best help her address her research question.

Given the specific aspect of feedback “trainees' perceptions of the feedback they receive” she aims to study and the social aspects of the educational alliance theoretical framework she is using to underpin her study, she opts for qualitative methodology because it could yield richer information about the feedback experience than quantitative methodology. Owing to her lack of experience in qualitative research, she reaches out to a qualitative research expert at her institution for advice and potential collaboration. Together, they plan the key research steps, such as sampling, data collection, analysis, and reporting. They conclude that semistructured interviews would be the best data collection method and discuss with the members of the CNPD to have multiple sites involved in study recruitment.

## Case 2: Procedural Skill Development

### Case Vignette

The Dean of Assessment at a medical school has received evaluation data from graduates who felt underprepared in hands-on medical procedures. She noticed that lumbar punctures are one of the procedures highlighted in the survey. She meets with the Neurology Clerkship Director, asking him to develop and evaluate an educational program for teaching bedside lumbar puncture to the neurology clerkship students.

The Neurology Clerkship Director is aware that it would not be feasible in the clinical environment for all clerkship students to practice or perform actual bedside lumbar punctures, given the variability of clinical experiences across clinical sites. He wonders if, in the process of finding a solution to the specific problem at his institution, there is a broader opportunity to conduct an educational research project on lumbar puncture instruction to provide insights for other neurology clerkship programs to train their students in this procedural skill.

### Conceptual Framework

#### Define the Educational Problem

The first step is to define the problem. In thinking about the phenomenon of procedural skill development, he remembers that lumbar puncture is a skill described as an objective in the AAN's Core curriculum guidelines for a required clinical neurology experience.^5^ Thus, this problem is relevant for neurology clerkships nationwide and to educators facing similar challenges in providing opportunities for procedural skill development.

The next step is to identify what is involved in teaching the procedural skill of lumbar puncture. In contrast to the feedback case above, we can assume that there would be less deviation in how we define what constitutes an effective lumbar puncture (i.e., performing an effective lumbar puncture is less context dependent because there is more agreement around the steps required to perform a lumbar puncture and determining when it is effective).

Nevertheless, questions around lumbar puncture skills training can be formulated in several ways: what are the perceptions of students on the need to learn lumbar punctures? What are the perceptions of the faculty on the need to teach the skill to students vs resident trainees? What are the most common aspects of the procedure where the students encounter difficulties? What is the rate of knowledge decay around procedural skills? What effective instructional methods can be used to train many students on lumbar punctures? What is the best way to assess successful attainment of the lumbar puncture skill?

He decides to concentrate on developing an instructional program to teach students lumbar puncture and assess their skill in a controlled setting, given the variability of exposure in the clinical environment.

#### Explore the Scholarly Conversation

The Neurology Clerkship Director reviews multiple research articles about lumbar puncture as a procedural skill and various instructional strategies used to teach and assess it.

Because he is focused on instruction and further on how to plan instruction systematically for a large number of participants, he finds good alignment with his research goals and a study published in *Neurology*^6^ that described simulation-based instruction in lumbar puncture to internal medicine and neurology residents.

#### Construct the Research Question(s) and Select the Theoretical Framework(s)

After defining the problem and its relevance and reviewing the literature on lumbar puncture instruction, he searches for theoretical frameworks to underpin his study. The simulation curriculum he plans to implement relies on simulation-based mastery learning (SBML) principles. SBML integrates 3 learning theories: behavioral learning theory, constructivist learning theory, and social cognitive learning theory,^7^ in addition to deliberate practice.^8^ He sees that behavioral learning theory in SBML is also associated with prolonged skill retention after learning and has been shown to improve advanced cardiac life support skills even after a 1-year delay.^9^

Thus, while SBML has informed his lumbar puncture curriculum on the immediate acquisition of skills, using SBML to develop enduring lumbar puncture skills has not yet been explored. To fill this knowledge gap, he decides to study his curriculum by assessing not only immediate improvements in students' skills in lumbar puncture but also reassessing them in another simulation-based assessment after a delay of 3 months.

#### Choose the Appropriate Methodology and Methods for the Research Question(s)

Now that he has chosen an instructional program to develop students' lumbar puncture skills informed by the theoretical framework of SBML and deliberate practice, he will assess the immediate and retained skills using a simulation-based assessment with a checklist, which represents a quantitative methodology. He starts planning his lumbar puncture simulation, learner assessments, and program evaluation.

## Case 3: Lecture Development

### Case Vignette

The preclinical Neuroscience Course Director meets with the Neurology Vice-Chair for Education to share faculty evaluation data from students describing low satisfaction with the neurology faculty lecture sessions for the neuroscience block. They discuss potentially developing a faculty development program to help improve the lecturing skills of the faculty.

While finding a solution to the specific problem at their institution, they wonder whether there might be a broader opportunity to conduct an educational research project on faculty development for preclinical neuroscience course teachers to provide insights to other institutions on developing and implementing lecturing skills.

### Conceptual Framework

#### Define the Educational Problem

The first step is to define the problem. In thinking about lecturing skills, the Course Director realizes that she first has to review the goals and objectives of the neuroscience course and determine which objectives map to each of the faculty lectures. This way, she has specific information about what each lecture should achieve.

Having a clear idea of the objectives for each lecture allows her to determine what it means to have an effective lecture anchored in objective data and not solely based on subjective perceptions of learners. She also has to consider the experience of the faculty giving the lecture (i.e., go beyond the student evaluations) to assess if the issue is about the lecture content, the content delivery, or the misalignment of educational expectations.

When thinking about an approach to the problem, she comes up with several questions: what is the lecture content? Is the content aligned with prior sessions and course learning objectives? How are the lectures delivered (e.g., recorded, live, or hybrid)? What factors influence the perceived benefit of the lectures, for example, timing, required attendance, and lecture length? Is there a misalignment between the educational expectations of faculty and students?

To assess the transferability of her findings to other contexts outside of her institution, she reaches out to several neuroscience course directors to ask if they have experienced similar problems with the lecture portions of the neuroscience course. She finds it a common problem for others and thus relevant to address in a research project.

#### Explore the Scholarly Conversation

The Course Director identifies considerable debate in the literature regarding the adequacy of student evaluations of teaching used solely for definitive faculty assessment.^10^ This strengthens the idea to focus her study on the development and implementation of a program that involves restructuring the lectures themselves (content and methods of delivery) rather than the perceptions of faculty teaching by learners or the faculty lecturing skills (which was the original intent of the initiative).

She then reviews the literature to understand the theoretical frameworks that could be used to inform her study. She reads with interest the “The Future of the Lecture in Neurology Education” article,^11^ which describes different learning theories that could help inform her study design and implementation.

#### Construct the Research Question(s) and Select the Theoretical Framework(s)

After defining the problem and its relevance and literature review, she decided that the study would focus on developing a system to structure the lecture content and delivery across a neuroscience course to align and reinforce the different course objectives.

In designing the program, she draws from cognitive load theory^12^ to inform the structural aspects of the lecture, such as timing, lecture content (e.g., using clinical cases as schemas to simplify content, lecture over image-based slides rather than text-based to minimize conflicting processing of information), and content delivery sequencing. She also informs her implementation plan using design-based research principles.^13^

#### Choose the Appropriate Methodology and Methods for the Research Question(s)

Once the Course Director has decided to develop a structure to modify lectures in a neuroscience course informed by cognitive load theory and underpinned by principles of design-based research, she goes forward with a program evaluation plan to judge and plan improvements for her curriculum redesign project.^14^

## Discussion

Substantial time and effort should be dedicated to the questions: what are we doing? Why are we doing it? How do we plan to do it? before embarking on an educational research study. As highlighted in this article, building a conceptual framework ensures that a research study is adequately conceptualized, supported, and rigorously designed.

As illustrated in the cases above, the groundwork underpinning a study involves clearly defining the problems being addressed through educational research; situating the research within a broader scholarly conversation, including defining how the research study will contribute to the field by a clear outline of the gap in the literature it intends to fill; formulating specific research questions; deciding on theoretical frameworks or models informed by a careful review of the literature; and finally choosing the appropriate methodology and methods for the research questions.

The examples demonstrate how educational research questions commonly originate from the local learning environment, derived from real-life concrete educational problems. This pragmatic approach allows for the research to have practical applications. Still, it can also rush the process by pushing educators to find a solution before adequately thinking about the problem beyond the local context and how to study it through rigorous educational research with the goal of having a broader relevance in the field. In addition, education research often settles on studies of skill or behavior-based outcomes without asking why and how these outcomes occur. The solutions to local educational problems are also often too narrow and context specific (i.e., only applicable to the specific educational problem at the institution). Developing educational scholarship should strive to move beyond local education quality improvement projects and aim to join a broader scholarly conversation, which gives it more relevance and enhances transferability to other contexts beyond the local settings.^15^

Using the problem-gap-hook heuristic^3^ is a helpful way to organize the description of the conceptual framework of the study and facilitates intentionally thinking about them in the early steps of the research process. A clear problem-gap-hook description should anchor the first paragraph or two of the introduction section of education scholarship papers.^16^ The scientific story starts in the introduction and must be brought to a close in the discussion section.^17^ This means that the conceptual framework(s) used to provide rationale for the study need to be clearly described in the introduction, with a closing of the loop linking them to the results in the discussion section.

Authors often reach out to peers, in addition to the literature review, to assess the potential transferability of prospective study findings to contexts outside of their own. Multiple educational forums for idea generation and refinement are available in the neurology field, such as the Consortium of Neurology Clerkship Directors and the CNPD. In addition, the education sessions at neurology professional society meetings are valuable opportunities to discuss and start new collaborations to strengthen the scope and reach of potential education research studies.

In summary, building a solid conceptual framework lays the groundwork to embark on the arduous work of developing and carrying out an educational research study. The critical steps of this groundwork involve defining the educational problem being addressed, the relevance of the problem beyond the local context, a careful review of the literature to understand the scholarly conversation around the topic, formulation of clear research questions, and underpinning theoretical frameworks that inform the study, and finally choosing the appropriate methodology and methods to answer the research questions with rigor. We can be reassured that we are on the right path if we deliberately lay a solid foundation for our future educational research work.

## Glossary

AAN: American Academy of Neurology
CNPD: Consortium of Neurology Program Directors
PD: program director
SBML: simulation-based mastery learning
